# Nusbiarylins Inhibit Transcription and Target Virulence Factors in Bacterial Pathogen *Staphylococcus aureus*

**DOI:** 10.3390/ijms21165772

**Published:** 2020-08-11

**Authors:** Adrian Jun Chu, Yangyi Qiu, Rachel Harper, Lin Lin, Cong Ma, Xiao Yang

**Affiliations:** 1 Department of Microbiology, Faculty of Medicine, The Chinese University of Hong Kong, Prince of Wales Hospital, Shatin, Hong Kong; adrianjunchu@cuhk.edu.hk (A.J.C.); rachelharper@cuhk.edu.hk (R.H.); linlin@cuhk.edu.hk (L.L.); 2 State Key Laboratory of Chemical Biology and Drug Discovery, Department of Applied Biology and Chemical Technology, The Hong Kong Polytechnic University, Hung Hom, Hong Kong; yangyi.qiu@connect.polyu.hk

**Keywords:** nusbiarylin, *Staphylococcus aureus*, MRSA, drug discovery, antimicrobial agent, antibiotic

## Abstract

The emergence of multidrug resistance in the clinically significant pathogen *Staphylococcus aureus* is a global health burden, compounded by a diminishing drug development pipeline, and a lack of approved novel antimicrobials. Our previously reported first-in-class bacterial transcription inhibitors “nusbiarylins” presented a promising prospect towards the discovery of novel antimicrobial agents with a novel mechanism. Here we investigated and characterised the lead nusbiarylin compound, MC4, and several of its chemical derivatives in both methicillin-resistant *S. aureus* (MRSA) and the *S. aureus* type strains, demonstrating their capacity for the arrest of growth and cellular respiration, impairment of RNA and intracellular protein levels at subinhibitory concentrations. In some instances, derivatives of MC4 were also shown to attenuate the production of staphylococcal virulence factors in vitro, such as the exoproteins α-toxin and Panton–Valentine Leukocidin (PVL). Trends observed from quantitative PCR assays suggested that nusbiarylins elicited these effects possibly by acting via but not limited to the modulation of global regulatory pathways, such as the *agr* regulon, which coordinates the expression of *S. aureus* genes associated with virulence. Our findings encourage the continued development of more potent compounds within this novel family of bacterial transcription inhibitors.

## 1. Introduction

*Staphylococcus aureus* is an opportunistic Gram-positive pathogen of high clinical significance, responsible for skin, soft tissue, respiratory and blood infections with a wide spectrum of severity [1]. The widespread prevalence of multidrug-resistant *S. aureus*, be it hospital-acquired (HA-MRSA) or community-associated (CA-MRSA), presents a serious global challenge to public health as infections increasingly fail to respond to existing antibiotic treatments [2]. The continued discovery of antimicrobial agents with novel mechanisms of action is therefore vital to the provision of alternatives in the clinical management of infectious diseases caused by resistant organisms.

### 1.1. The Interaction between Bacterial Transcription Factors NusB-NusE as a Drug Target

A crucial step in the central dogma of molecular biology, bacterial transcription, serves as a target for developing novel antimicrobials, where two drugs—the broad-spectrum rifamycin and the narrow-spectrum anticlostridial fidaxomicin are currently in use [3]. Transcription is driven by the multi-subunit RNA polymerase (RNAP), a process regulated through the interaction with several transcription factors. The N-utilisation substances (Nus) factors belong to a key family of transcription factors comprised of NusA, NusB, NusE (ribosomal protein S10), NusG, ribosomal protein S4 and more recently discovered SuhB, all of which are involved in the transcription of bacterial ribosomal RNA (rRNA) operons, folding, ribosome biogenesis, as well as coupling cellular transcription and translation processes [4,5,6,7,8].

**Figure 1:**
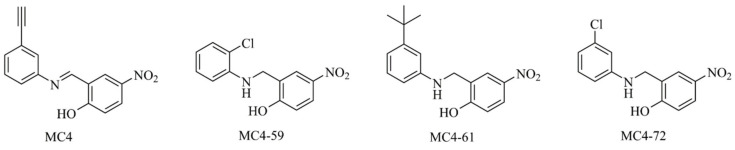
The chemical structures of selected nusbiarylins studied in this work. MC4, the lead compound, and its derivatives MC4-59, MC4-61 and MC4-72 [11,12,13].

The formation of the NusB-NusE heterodimer is a crucial step in the bacterial rRNA operon transcription complex, which binds to *boxA* sequence elements of rRNA operons upstream of 16S and 23S rRNA, presenting a potential druggable target for the development of novel antimicrobials [9,10]. In our earlier attempts, we discovered a novel class of antimicrobials targeting this specific protein-protein interaction (PPI) [11,12]. The hit compound MC4 represents the first-in-class inhibitor of bacterial rRNA transcription [13]. Subsequently, chemical derivatives of MC4 have been synthesised and biologically evaluated, showing good antimicrobial activity with minimum inhibitory concentrations (MICs) of 1–2 μg/mL against pathogens of clinical significance [13]. Based on the target protein NusB and the biaryl chemical structure, MC4 and its derivatives were collectively named as nusbiarylins [11,12,13]. Notably, our selection of nusbiarylins also showed moderate to good antimicrobial activity against *Staphylococcus aureus* type strains, as well as both representative hospital-acquired (HA-) and community-associated (CA-) Methicillin-resistant *S. aureus* (MRSA) strains. To further characterise the antistaphylococcal profile of our select nusbiarylins—MC4 and its derivatives MC4-59, MC4-61 and MC4-72 (Figure 1)—so chosen for their good range of MICs (2–8 μg/mL), virulence-associated parameters were chosen and investigated to establish a more comprehensive picture.

### 1.2. Toxins as Virulence Factors in S. aureus

The ability to monitor environmental cues and instigate specific response patterns in metabolism and gene expression is a key prerequisite that underlies the fitness of *S. aureus* spp. [14]. One such mechanism in place is the *agr* (accessory gene regulator) quorum-sensing architecture, that drives cellular fitness and consequentially the capacity of staphylococci for opportunistic pathogenesis [14]. The ubiquitous staphylococcal *agr* locus is required for optimal post-log phase expression of the secretory proteins [15]. Staphylococcal alpha haemolysin (or Hla, alpha-toxin, α-toxin) is one such exoprotein key to *S. aureus* disease, where expression of the *hla* gene is induced at 37 °C at the mid-to late exponential phase [16]. Once induced, α-toxin is rapidly released into the extracellular environment, accounting for up to 33% of total protein in culture with only ≤1% α-toxin remaining intracellular [17,18]. A major cause of cellular injury, α-toxin is cytolytic to a wide range of human cell types and is a dominant virulence factor in CA-MRSA [19,20].

Panton–Valentine Leukocidin (PVL), on the other hand, is a virulence factor thought to have originated from methicillin-susceptible *S. aureus* (MSSA) that forms pores in the membranes of polymorphonuclear leukocytes (neutrophils) through the synergistic action of subunits LukS-PV (encoded by *lukS-PV*) and LukF-PV (encoded by *lukF-PV*), and is indicated in cases with severe inflammatory lesions, skin and soft tissue necrosis and serious systemic infections [21]. PVL is consistently associated with pathogenesis over colonisation, and is more prevalent in dermatopathological incidences than invasive staphylococcal disease [22]. Whilst PVL carriage accounts for 0.9–11.6% of *S. aureus* infections in high-income countries such as the UK, France, Korea and Singapore, it has been reported that PVL-producing staphylococci (both MSSA and MRSA) are predominantly community-acquired [23].

### 1.3. Global Regulatory Picture of agr and Associated Pathways in S. aureus

As aforementioned, the expression of α-toxin and PVL in *S. aureus* is regulated by the *agr* locus, which is the best-studied quorum-sensing system tightly-linked to toxin expression [24,25]. The *agr* operon both upregulates and downregulates transcription of a plethora of toxins, exoproteins and virulence determinants, and is comprised of two divergent promoters P2 and P3, the latter of which is for the primary transcript and effector molecule RNAIII (which also contains a coding sequence for *hld*—δ-toxin or delta-haemolysin) [26]. P2, on the other hand, oversees the expression of proteins AgrB, -D, -C and -A (*agrBDCA*), respectively, which are collectively known as the primary transcript RNAII and have autoinducing (AgrD and AgrB—a cyclic autoinducing peptide and its exporter), signal-transducing (AgrC, a membrane histidine kinase), autoregulatory (AgrA) and ultimately quorum-sensing functions [27,28]. *Agr* can therefore regulate the expression of cytolytic exoproteins, such as phenol-soluble modulins (PSMs), PVL, α- and δ-toxins, toxic shock syndrome toxins (TSST), serine protease (*sspA*) and staphylococcal protein A (*spa*), through both direct binding of downstream genes or indirectly through regulating its own effectors or via other regulators [29].

**Figure 2:**
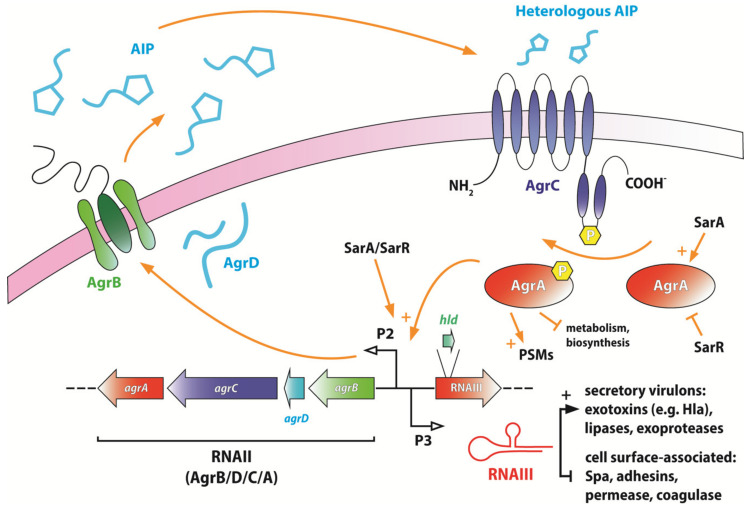
Schematics of the *agr* regulon in *S. aureus* and its relationship with key determinants of virulence and cell maintenance. AIP: autoinducing peptide; PSMs: phenol-soluble modulins. Figure modified from [36].

Albeit a global regulator, the activity of *agr* is itself also under the mediation of other regulators, such as SarA/SarR and CodY [30,31]. The staphylococcal accessory regulator (*sar*) locus encompasses the transcripts *sarA*, *sarB* and *sarC*, where SarA and its repressor homologue SarR are essential for modulating toxin production through their control over the *agr* P2 and P3 promoters [32,33]. Singular target virulence genes being under the dynamic influence of multiple “cross-talking” regulators has been described as an energetically-favourable mechanism [34]. The above network of regulons presents viable targets for chemotherapeutic agents to achieve virulence attenuation (Figure 2) [28,35].

### 1.4. Aim

The characterisation of α-toxin and PVL expression, as well as that of their associated regulatory pathways in *S. aureus* treated with chemotherapeutic compounds, are therefore presented as appropriate parameters and serve as objective means to assess the antimicrobial profile of our panel of nusbiarylins (listed in Section 1.1), which in turn may also shed light into their mechanism of action in molecular studies. In this study, we examined the performance of MC4, MC4-59, MC4-61 and MC4-72, previously reported for good antimicrobial activities against various *S. aureus* spp., with the use of both cellular and molecular techniques to provide mechanistic insights. Experiments were devised to ascertain the antimicrobial profile of the selected nusbiarylins as indicated in our previous reports by assessing their capacity to attenuate staphylococcal virulence through toxin expression, and their resulting genotypic and phenotypic responses compared to representative drugs already in clinical use.

## 2. Results

### 2.1. Minimum Inhibitory Concentrations (MICs)

Our selection of nusbiarylins was comprised of the previously published lead compound MC4 and its derivatives MC4-59, MC4-61 and MC4-72 (Figure 1; MC4 = Compound 14 in [11] and Compound 1 in [12]; MC4-59 = Compound 27 in [12]; MC4-61 = Compound 23 in [11,12]; MC4-72 = Compound 28 in [12]) were tested against *S. aureus* type strains ATCC^®^ 25923, ATCC^®^ 29213, as well as MRSA lineages USA300 (community-acquired) and ST22 (hospital-acquired) to determine their MICs as stipulated by the Clinical & Laboratory Standards Institute (CLSI) [6,11,12,13,37]. In this study we revisited the MIC screening to verify the values for the same strains when cultured in the more staphylococci-optimised tryptic soy broth (TSB) in preparation for the downstream characterisation assays, rather than the standard Mueller-Hinton Broth (MHB) used for MIC tests in the previous studies (MIC data for MC4, MC4-59, MC4-61 and MC4-72 in MHB-cultured 25923 and 29213 had previously been published as Compounds 1, 27, 23 and 28, respectively, in Table 3 in [12]; MIC data for MC4-59, MC4-61 and MC4-72 in MHB-cultured USA300 and ST22 had previously been published as Compounds 27, 23 and 28, respectively, in Table 5 in [12]; MIC data for MC4 and MC4-61 in MHB-cultured 25923, 29213, USA300 and ST22 had previously been published as Compounds 14 and 23, respectively, in Figure 2B in [11]). MIC values of MC4, MC4-59, MC4-61 and MC4-72 against TSB-cultured type strains and MRSA strains were within ±2–4 folds of MHB-cultured set-ups. Overall, the MC4 compounds showed good antimicrobial potency against the staphylococcal strains tested, achieving those of the cell wall synthesis inhibitor vancomycin and oxacillin, which are clinically available (Table 1).

### 2.2. Time–Kill Kinetics and Central Metabolism

**Figure 3:**
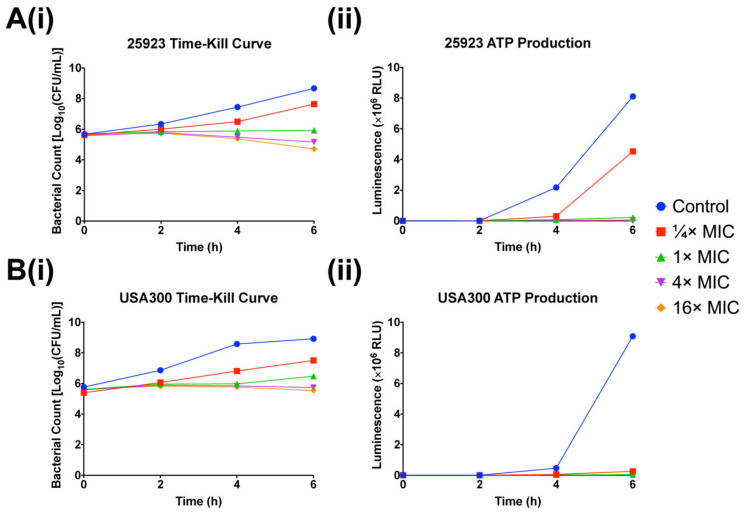
Effects of MC4 on the (**i**) time–kill kinetics and (**ii**) ATP production of (**A**) *S. aureus* type strain ATCC^®^ 25923 and (**B**) CA-MRSA strain USA300 when challenged at ¼ ×, 1 ×, 4 × and 16 × MIC in TSB media. Experiments were repeated in triplicates. 25923: *S. aureus* ATCC^®^ 25923; USA300: CA-MRSA strain USA300.

MC4 was added to *S. aureus* ATCC^®^ 25923 and USA300 at concentrations relative to their respective MICs, where the time- and dose-dependent relationship between nusbiarylins and bacterial growth and viability were established through time–kill kinetics and ATP production assays. Overall, MC4 was bacteriostatic against both *S. aureus* ATCC^®^ 25923 and USA300 over the course of 6 h, with only slight decreases in CFU counts at the highest concentrations towards the end of the assay (Figure 3). Time–kill kinetics of MC4 against *S. aureus* ATCC^®^ 25923 cultured in MHB had previously been published (as Compound 1, Figure 6 in [12]), which was consistently bacteriostatic with the TSB-cultured set-up reported in this study.

The arrest of cellular respiration is a hallmark characteristic of effective antibiotics [38]. The rate of ATP production of both *S. aureus* strains was monitored simultaneously as the time–kill kinetics assay proceeded using the same experimental setup, where samples were taken at identical time points for readings. Significant decrease in ATP production rate was observed beginning at ¼ × MIC of MC4 compared to untreated control, with further suppression as concentrations increased. Taking into account of the role of MC4 as a transcription inhibitor, the results were in concordance with previously reported trends displayed by the class drug rifampicin, where bacterial respiration rate is inversely correlated to the drug efficacy of antimicrobials [39,40].

### 2.3. Exotoxin Release

**Figure 4:**
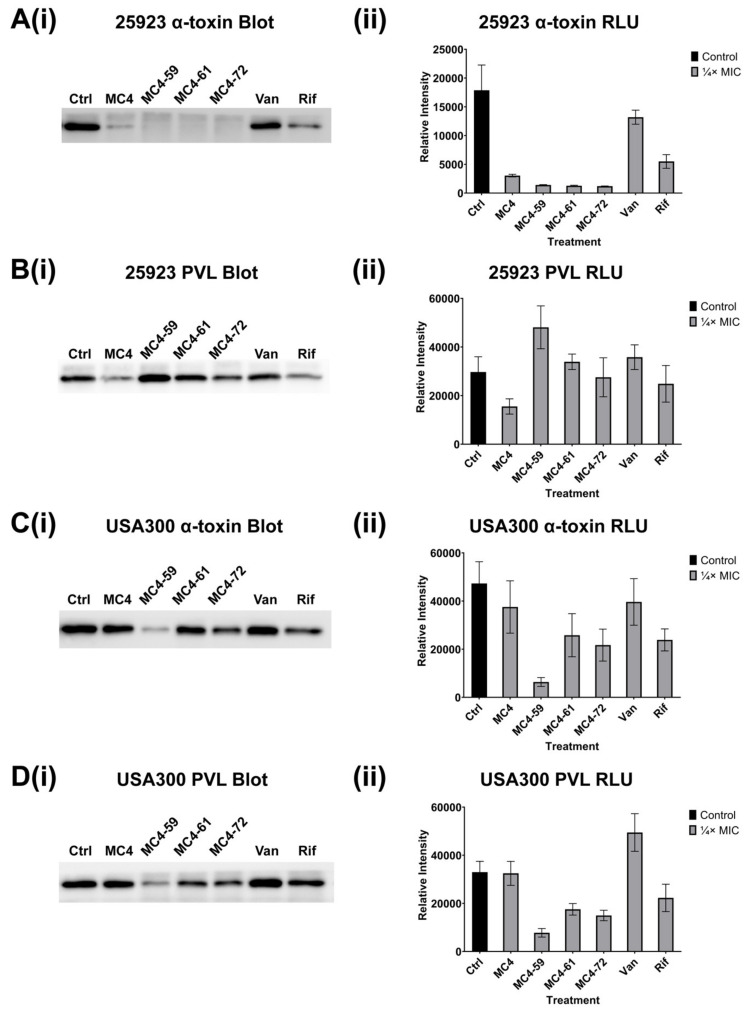
Western blot analysis of the effects of selected nusbiarylins in the expression of (**A**) α-toxin and (**B**) Panton–Valentine Leukocidin (PVL) in *S. aureus* ATCC^®^ 25923; (**C**) α-toxin and (**D**) PVL in MRSA USA300 at subinhibitory concentration, presented as (**i**) blot image and (**ii**) relative intensity. Each experiment was performed in triplicates and representative figures shown. 25923: *S. aureus* ATCC^®^ 25923; USA300: CA-MRSA strain USA300; Van: vancomycin; Rif: rifampicin.

The effects of our panel of nusbiarylins on the levels of α-toxin and PVL production in *S. aureus* ATCC^®^ 25923 and USA300 were assessed by immunoblotting exoproteins secreted into supernatants harvested overnight cultures grown in the presence of sub-MIC compounds and control drugs (vancomycin and rifampicin). Proteins separated into bands by gel electrophoresis were blotted onto membranes and incubated with corresponding antibodies to both toxins. Signals from bands were imaged, their relative luminescence quantified and both data presented side-by-side (Figure 4). The results showed that in *S. aureus* ATCC^®^ 25923, MC4 and especially its derivatives significantly suppressed the release of α-toxin into the extracellular milieu compared to vancomycin and, to a certain extent, rifampicin (Figure 4A). Although MC4 and rifampicin also saw some response in decreasing PVL expression in the type strain, MC4 derivatives, however, did not appear to have meaningfully attenuated PVL levels in harvested supernatant (Figure 4B). In USA300, MC4-59 appeared to have elicited a significant decrease in both α-toxin and PVL expression, but a different trend for MC4 and other derivatives. α-toxin levels did not appear to have been substantially reduced by MC4-61 and MC4-72 compared to rifampicin and even, in the case of MC4, vancomycin (Figure 4C). This trend was also observed in PVL expression, but the level of toxin attenuation was more pronounced for MC4-61 and MC4-72 compared to that of vancomycin (Figure 4D).

Overall, the observed trend where vancomycin had largely little to no effect on the production of PVL in stationary phase *S. aureus* was consistent with previously reported findings [41,42]. The similar lack of any significant inhibitory effect was also found in USA300 for both α-toxin and PVL, and was consistent with observations previously reported in MRSA strains [43]. Nusbiarylins, in contrast, appeared to be at a similar level to the performance of the class drug rifampicin and, for some of the MC4 derivatives, even appeared to have more markedly attenuated toxin expression in both strains.

A plausible explanation for the occasionally observed nonspecific binding of α-LukS-PV primary antibodies to the membrane regions at immediately higher molecular weights than LukS-PV throughout the Western blots is the presence of staphylococcal protein A (Spa), which may be alleviated through DEPC treatment [44]. In this case, the interference was mitigated through horizontal trimming of the membrane to ensure only our target band size region is exposed to primary antibody incubation.

### 2.4. Rabbit Erythrocyte Haemolysis

The effects of select nusbiarylins on the in vitro production of α-toxin in *S. aureus* strains were assessed by the spectrophotometric analysis of haemoglobin release following exposure of the rabbit red blood cells (RBC) to the haemolytic agents. Culture supernatants containing secretory exotoxins harvested for the Western blot analysis in Section 2.3 were also used for the rabbit RBC lysis assay. Following preparation of packed and PBS-washed RBC suspension, erythrocytes were incubated with serially diluted supernatant samples and the absorbance measured at the end point.

**Figure 5:**
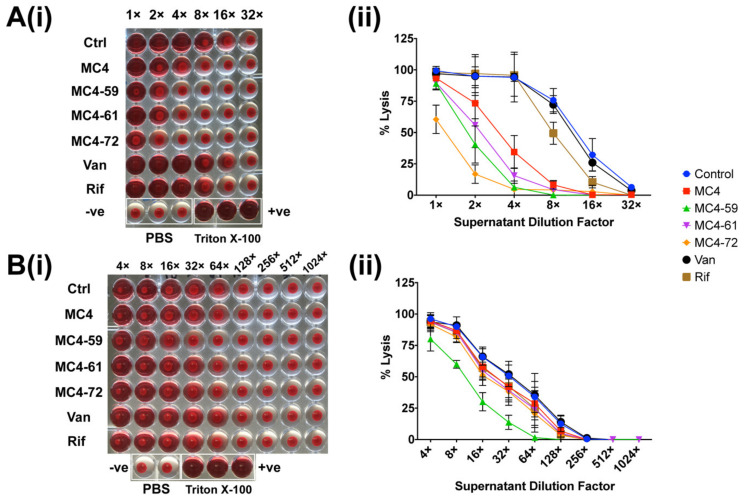
Effects of selected nusbiarylins on α-toxin-mediated lysis of rabbit red blood cells (RBCs) in (**A**) *S. aureus* ATCC^®^ 25923 and (**B**) MRSA USA300 (readings normalised with maximum as 100%) at subinhibitory concentrations, shown as (**i**) plate image and (**ii**) % lysis plot derived from absorbance changes. Supernatants from both drug-treated and untreated overnight bacterial cultures were serially diluted (× = folds diluted) and incubated with suspension of packed rabbit RBCs, with plain phosphate-buffered saline (PBS) as negative lysis control and 1% Triton X-100 as positive lysis control. Experiments were repeated in duplicates and representative data presented. Van: vancomycin; Rif: rifampicin.

The results showed that MC4-72 performed best in protecting RBCs from lysis by *S. aureus* ATCC^®^ 25923-derived α-toxin, whereas MC-59 performed best against USA300-derived α-toxin (Figure 5). In the *S. aureus* type strain, MC4-72, MC4-59 and MC4-61 were able to confer decreasing degree of toxin attenuation (Figure 5A). From there onwards, there was a significant performance gap in the mitigation of RBC lysis between MC4 and rifampicin, which remained completely haemolytic even when the supernatant was diluted by four-fold. Vancomycin had no apparent effect on α-toxin production compared to untreated controls in both *S. aureus* ATCC^®^ 25923 type strain and USA300.

In USA300, all supernatants used required 64- up to 1024-fold of serial dilution before attaining total absence of haemolysis, which is in line with its reputation as a hypervirulent strain. MC4-59 appeared to markedly attenuate α-toxin levels in USA300, resulting in observably less RBC lysis in comparison with other nusbiarylins, vancomycin, rifampicin as well as the untreated control (Figure 5B). All observations were in concordance with those in the Western blot analysis (Section 2.3), and greatly complemented the findings—particularly for *S. aureus* ATCC^®^ 25923—by offering enhanced resolution on the differences in performance between the MC4 derivatives.

### 2.5. Real-Time qPCR of Virulence-Associated Gene Expression

Cells of *S. aureus* ATCC^®^ 25923 and MRSA strain USA300 were treated with MC4 and the class drug rifampicin at subinhibitory concentrations, together with the untreated control. The levels of total DNA across samples set-up for qPCR analysis were first quantified to ensure conformity among harvests. Purified RNA samples were then transcribed into complementary DNA (cDNA) which acted as templates for subsequent real-time quantitative PCR (qPCR) assays.

#### 2.5.1. Effects on rRNA Complex and Implications on the Housekeeping Genes

**Figure 6:**
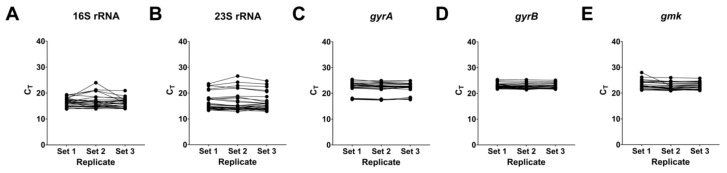
Distribution of C_T_ values of some commonly used “housekeeping” genes. Relative qPCR assay signal levels of (**A**) 16S rRNA, (**B**) 23S rRNA, (**C**) *gyrA*, (**D**) *gyrB* and (**E**) *gmk* in *S. aureus* ATCC^®^ 25923, 29213 and MRSA strain USA300. Data irrespective of strains or treatment groups were all plotted together as *y*-values and segregated by their corresponding replicate sets on the *x*-axis. Experiments were performed in replicates.

A defining mechanism of action of nusbiarylins is the inhibition of nascent Nus-regulated essential PPIs within the wider transcription machinery and perhaps beyond rRNA, which invites the question whether the expression levels of classical housekeeping genes commonly used in qPCR and sequencing but are integral to the bacterial rRNA operon transcription complex—such as 16S rRNA, could feasibly serve as valid points of reference [45]. To address this issue, qPCR assays were first performed by probing the distribution of both the drug-treated and untreated signal levels of 16S rRNA, 23S rRNA, DNA gyrase A (*gyrA*), DNA gyrase B (*gyrB*) and guanylate kinase (*gmk*) in the *S. aureus* type strains and MRSA strain USA300, all of which had previously been indicated or used in gene expression quantitation studies [46,47]. The results suggested that both rRNA subunits showed high variation in the disposition of C_T_ values across the treatment and replicates, indicating that their expression had been affected by the presence of subinhibitory levels of rifampicin and MC4 during growth (Figure 6A,B). In contrast, the C_T_ values of the more metabolism- and maintenance-related genes *gyrA*, *gyrB* and *gmk* were more similar across the experimental replicates, suggesting they could serve as appropriate housekeeping genes due to their relative indifference towards the transcription inhibitors used (Figure 6C–E). When considering that C_T_ values are binary logarithms since DNA strands double after each cycle, the larger the differences therein implies a higher degree of susceptibility to inhibitory effects attributable to the use of rRNA- and RNAP-targeting agents. This method offered a quick yet informative preview into the effects of nusbiarylins, thus ensuring that unsuitable housekeeping genes were avoided as potential points of reference were given consideration in qPCR-based expression studies.

#### 2.5.2. Quantitative PCR

Given the findings from Section 2.5.1 and relevant implications from literature, real-time qPCR assays were performed using *gyrB* as reference gene, with each treatment group normalised against an untreated control [48]. Relative expression levels of key genes associated with virulence, namely *agrA* (AgrA), RNAII (encoding the AgrB/D/C/A regulon), RNAIII (toxin-mediating regulon) and *sarA* (SarA/R regulon), as well as the secretory exoproteins under their modulation *spa* (Spa), *hla* (α-toxin) and *lukS* (LukS subunit of PVL), were probed with SYBR Green system and results extrapolated using the ΔΔC_T_ method (Figure 6).

**Figure 7:**
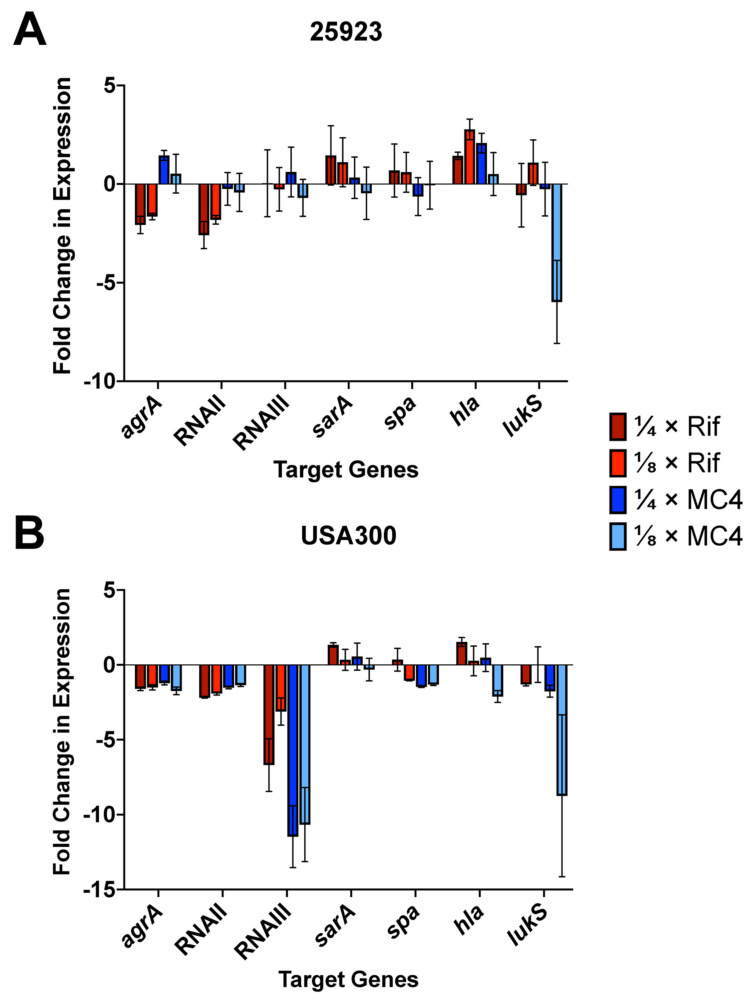
Effects of subinhibitory levels of MC4 and rifampicin on the relative expression of virulence-associated genes in (**A**) *S. aureus* ATCC^®^ 25923 and (**B**) MRSA strain USA300. Probed transcripts belong to the staphylococcal SarA/SarR (*sarA*) and *agr* regulons (*agrA*, RNAII, RNAIII), as well as clinically significant exotoxins Protein A (*spa*), α-toxin (*hla*) and PVL (*lukS*). Fold change values (2^−^*^ΔΔ^^CT^* for upregulation; 1/−2^−^*^ΔΔ^^CT^* for downregulation) were extrapolated from ΔΔC_T_ calculations. Experiments were performed in triplicates. 25923: *S. aureus* ATCC^®^ 25923; USA300: CA-MRSA strain USA300; Rif: rifampicin.

In general, the expression of RNAII and RNAIII in USA300 were more downregulated following the addition of both rifampicin and MC4 when compared to *S. aureus* ATCC^®^ 25923. The relative lack of change in USA300 SarA expression treated with rifampicin and MC4 suggested that the drugs did not significantly promote *sarA* and by extension the phosphorylation of AgrA, which may also explain its more repressed *agrA* levels (Figure 7B). Compared to untreated control, the impact of the transcription-inhibiting agents on the expression of genes downstream of RNAIII, such as the surface-associated staphylococcal protein A (Spa), as well as the secretory exoproteins in USA300 such as α-toxin (Hla) and PVL (LukS) was limited. This result complemented the Western blot observations above, which also showed no significant difference in the levels of α-toxin and PVL in USA300 culture supernatants following treatment at ¼ × MICs.

For PVL under ⅛ × MIC of MC4, however, there appeared to be sharp downregulation in expression in both the type strain *S. aureus* ATCC^®^ 25923 and USA300 when compared to the equivalent concentration of rifampicin and untreated control, suggesting a role for dosage and that lower doses may elicit more profound inhibitory effects on certain types of toxins. Apart from rifampicin-treated downregulation of RNAII and its autoinducing effector *agrA*, no apparent trends were observed for all probed virulence-associated genes following the use of MC4 in *S. aureus* when compared to both the class drug and untreated control (Figure 7A). A point of intrigue appeared to be the upregulated expression of *hla* in the type strain following the addition of rifampicin and to a lesser extent MC4.

## 3. Discussion

Overall, our findings supported the notion that the nusbiarylin lead compound MC4 and its chemical derivatives MC4-59, MC4-61 and MC4-72 were able to attenuate the release of staphylococcal α-toxin and PVL into the extracellular space compared to untreated control, which in turn protects host cells such as RBCs from injury and lysis. Notably, at least one or more nusbiarylins tested performed better in these aspects than vancomycin and even rifampicin in several occasions, despite not necessarily attaining significantly lower MICs in standard screening or eliciting a marked bactericidal response instead of being bacteriostatic at sub-MICs. Observations from qPCR also indicated that nusbiarylins were able to mediate responses in virulence-associated genomic pathways under the influence or modulation of global regulators.

At the molecular level, MRSA strain USA300 appeared more sensitive towards the addition of MC4 and rifampicin compared to the type strain *S. aureus*, where transcripts of the *agr* regulon appeared to respond more definitively to the presence of inhibitory agents. While the same could not be said for the type strain *S. aureus* ATCC^®^ 25923, this suggested that both transcription inhibitors may also possess the ability to act via alternate staphylococcal pathways not encompassed in our experimental design to ultimately achieve the toxin attenuation phenotype as demonstrated in the Western blots of culture supernatants. It must be noted that cultures harvested for qPCR analysis provided only a snapshot of the cellular response to MC4 and rifampicin at mid-log phase in order to accommodate for the aforementioned optimal expression window reported for “housekeeping” genes but not necessarily *agr*-mediated post-log phase toxin secretion, which may contribute to the limited response resolution observed compared to that of harvests from overnight stationary phase cultures in immunological-based assays.

Subinhibitory concentrations of antibiotics are considered to have strong selection pressure on target bacteria, which drives mutation rates in response to incoming antimicrobial agents [49]. Exposure to lower doses of antibiotics could provide time and opportunity for stressed bacteria to enable adaptation to the chemotherapeutic challenge, potentially even altering pathogenicity through genotypic and phenotypic variations [50]. Given that most studies into antimicrobials were based on the potency of compounds at subinhibitory levels, it is important to take note of hormesis, a concept describing the biphasic response of bacteria where low dosage could at times paradoxically induce virulence [51]. In that regard, Davies et al. proposed that, in light of previous studies suggesting how the transcription inhibitor class drug rifampicin both targets toxin secretion yet upregulates survival, it is possible that sub-MIC rifampicin could modulate bacterial genes through allosteric changes in complex macromolecular structures unique to different bound ligands, which in turn lead to differently altered affinity and sensitivity of macromolecular targets towards environmental conditions such as the availability of magnesium ions [51]. To that end, it could be suggested that differences in sensitivity and response shown in our qPCR observations between *S. aureus* type strain and USA300 treated by transcription-targeting nusbiarylins at sub-MICs reflected the vastness of this wider framework of thought, where its therapeutic implications remain to be further studied.

In this work, we demonstrated that our novel family of bacterial transcription inhibitor nusbiarylins, represented by the lead drug MC4 and its derivatives, was capable of virulence attenuation in *S. aureus* spp. through arresting metabolism, inhibiting cell proliferation, protecting host cells from lytic effects of toxins and eliciting downregulating effects on key determinants of cellular fitness and maintenance associated with staphylococcal disease. As an inhibitor of crucial transcription-related PPIs in bacteria [52], nusbiarylins act in non-lytic means which puts it in a more favourable light than the cell wall synthesis inhibitor vancomycin, a last resort drug with increasingly emergent resistance in *S. aureus* species. In the face of the global antibiotic crisis and to meet the urgent clinical needs for novel treatment options, our findings warrant further development and characterisation of similar antimicrobial compounds to rapidly expand the profile of this underutilised drug class.

## 4. Materials and Methods

### 4.1. Determining Minimum Inhibitory Concentrations (MICs)

The antimicrobial activities of all compounds used in this study were assessed by broth microdilution in accordance with the guidelines issued by the CLSI [37]. Test media used were cation-adjusted Mueller-Hinton broth (CA-MHB) (CM0405, Oxoid, Basingstoke, United Kingdom), or tryptic soy broth (TSB) (CM0129, Oxoid) when staphylococcal optimisation was required to determine appropriate dilutions for downstream assays. Serial dilutions in two-folds were performed for test compounds, ranging from 256 μg/mL to 0.25 μg/mL for the nusbiarylins (MC4 and its chemical derivatives), 64 μg/mL to 0.0625 μg/mL for vancomycin and oxacillin, and 2 μg/mL to 0.002 μg/mL for rifampicin. Bacterial inoculum was adjusted to ~5 × 10^5^ CFU/mL. Following 20 h of overnight incubation at 37 °C, results were recorded, and MIC was defined as the lowest concentration of antimicrobial compounds used with no visible growth in well plates. Experiments were performed in triplicates.

### 4.2. Time–Kill Kinetics

The dose- and time-dependent antimicrobial effects of MC4 on *S. aureus* strains under aerobic conditions were assessed by adapting from relevant CLSI guidelines [53]. Log phase staphylococci cells were suspended at ~1.5 × 10^6^ CFU/mL in TSB containing test compounds at predetermined concentrations (i.e., ¼×, 1×, 4× and 16 × MICs) along with untreated controls. Cultures were set-up at 37 °C with agitation at 175 rpm, and 20 μL samples were retrieved from each treatment group at 0, 2, 4 and 6 h and underwent 10-fold serial dilutions in sterile PBS. Diluted suspensions were plated onto Columbia blood agar plates and the number of viable bacterial colonies was counted and expressed as CFU/mL following overnight incubation at 37 °C. The experiment was performed in triplicates.

### 4.3. ATP Production Assay

Performed simultaneously as the time–kill kinetics, 20 μL samples were retrieved from the same cultures for each treatment group at the same time points (0, 2, 4 and 6 h) and added to white 96-well plates, after which equal volumes of reagents were added from the BacTiter-Glo™ Microbial Cell Viability Assay Kit (Promega, Madison, WI, USA) and quantified according to the manufacturer’s protocol. The experiment was performed in triplicates.

### 4.4. S. aureus Toxin Release

To investigate the effects of our panel of nusbiarylins on the levels of α-toxin and PVL production, immunoblotting of exoproteins harvested from culture supernatants was performed. *S. aureus* ATCC^®^ 25923 was chosen as the type strain control as *S. aureus* ATCC^®^ 29213 is negative for PVL expression [54]. USA300, the CA-MRSA clone with exceptional cellular fitness and epidemiological success, was used as the PVL-positive (*pvl*^+^) MRSA strain due to its profound clinical significance both inside and outside hospital settings [55,56]. α-toxin, LukS-PV and LukF-PV are all polypeptides of ~33 kDa in molecular weight, and LukS-PV was chosen as the probing target for the detection of PVL expression. Purified recombinant α-toxin and LukS-PV were included in each corresponding run as standards and positive controls.

Fresh colonies of both *S. aureus* ATCC^®^ 25923 and MRSA strain USA300 were attained following overnight incubation in LB agar. A starter culture of 50 mL in a 250 mL autoclaved conical flask was inoculated at OD_600_ 0.1 and allowed to grow to OD_600_ ~0.2 with agitation at 175 rpm, after which it was split into 6 mL aliquots. Cells were grown overnight in TSB supplemented with ¼ × MIC of MC4, MC4-59, MC4-61 and MC4-72, respectively, after which 1 mL aliquots from each 6 mL culture were centrifuged at 3000× *g* for 3 min and the supernatants containing the secretory exoproteins were retained. Samples not immediately used were stored at –20 °C until use in downstream assays. Supernatants were bound with loading dye, heat-denatured and loaded onto polyacrylamide gels for SDS-PAGE analysis, after which they were blotted onto 0.2 μM nitrocellulose membranes (#1620112, Bio-Rad, Hercules, CA, USA) and probed with antibodies targeting α-toxin and LukS-PV.

### 4.5. Western Blot

Samples were prepared by mixing 15 μL supernatant from the corresponding harvests above with 3 μL 6× loading dye, boiled for 10 min in 0.5 mL PCR capped strip tubes and loaded onto 12% polyacrylamide gels. Then, 50 ng of recombinant alpha haemolysin protein (ab233724, abcam, Cambridge, United Kingdom) with a predicted weight of 33 kDa, and 5 ng of recombinant *S. aureus* LukS-PV protein from *E. coli* (DAGB198, Creative Diagnostics, NY, USA) with a molecular weight of 32.465 kDa were added alongside each assay to serve as standards for visual reference. Samples were separated by SDS-PAGE, where bands were first stacked at 80 V for 15 min and then resolved at 150 V for 1 hr. Peptides were blotted to a nitrocellulose membrane (#1620112, Bio-Rad, Hercules, CA, USA) at 110 V for 1 hr, after which the membrane was blocked with 5% nonfat milk in TBST for 1 h and incubated overnight with primary antibodies which were either 1 μg/mL mouse monoclonal anti-alpha-haemolysin (ab190467, abcam, Cambridge, United Kingdom) or 0.5 μg/mL rabbit polyclonal anti-PVL LukS subunit (ab190473, abcam, Cambridge, United Kingdom) at 4 °C with agitation. Membranes were then incubated at room temperature with agitation with the corresponding HRP-conjugated secondary antibodies: 1:3000 goat anti-rabbit (ab97051, abcam, Cambridge, United Kingdom) or 1:5000 goat anti-mouse (ab205719, abcam, Cambridge, United Kingdom), flanked with TBST wash cycles in between. Bio-Rad Clarity™ Western ECL Substrates were used to develop the blots and visualised in a Bio-Rad ChemiDoc™ Touch system under the Chemiluminescence mode at manual exposure settings (Bio-Rad, Hercules, CA, USA). The experiment was performed in triplicates and representative data was presented.

### 4.6. Rabbit RBC Lysis

Supernatants harvested from the same overnight cultures set-up for the Western blot analysis of α-toxin and PVL expression were also used for the indirect assessment of exotoxin levels in treated *S. aureus* cells through determining haemolysis of rabbit erythrocytes. Haemoglobin released into plasma through exposure to lytic agents serves as a measurement for RBC lysis. Rabbit RBCs were packed by discarding the supernatant from thrice PBS-washed and centrifuged (200× *g* for 2 min) 1 volume of cell-phase rabbit blood. When a clear supernatant was achieved with no signs of haemolysis, a 3% *v*/*v* suspension of rabbit RBCs was prepared by mixing 1 volume of packed RBCs with 32 volumes of PBS. Due to the sensitivity of rabbit RBCs to concentrated levels of toxins in harvested culture supernatants, 20 μL samples were serially diluted on a 48-well plate and 180 μL of 3% rabbit RBC was added to each well. Then, 1% *v*/*v* Triton X-100 (2 μL diluted in 18 μL PBS) was included as a positive control as it can completely lyse the 3% RBC inoculum, while 200 μL PBS served as blank. The plate was incubated at 37 °C for 1 h and centrifuged at 2000× *g* for 3 min. The plate supernatants were transferred to a new plate in volumes of 100 μL per well, and the absorbance measured at a wavelength of 540 nm. Results were expressed as percentage haemolysis i.e., blank-normalised sample value/value from Triton-treated well × 100%. The experiment was performed in replicates.

### 4.7. Real-Time qPCR

Purified RNA harvested in the above cell content quantitation assay was used as templates to synthesise cDNA for qPCR assays using the High-Capacity cDNA Reverse Transcription Kit (Applied Biosystems, Foster City, CA, USA), the product of which was quantified with the Qubit™ ssDNA Assay Kit (Invitrogen, Carlsbad, CA, USA) using the Qubit 4 Fluorometer (Thermo Fisher, Waltham, MA, USA). qPCR primers used and their respective sources of design were shown in Table 2. Primers and cDNA templates were diluted appropriately according to the recommendations of the manufacturer of the 2X PowerUp™ SYBR™ Green Master Mix (Applied Biosystems). For 20 μL reactions, 10 μL of the 2X master mix was added to each well in a 0.1 mL-sized MicroAmp^®^ Fast 96-Well Reaction Plate (Applied Biosystems), followed by 2 μL of forward primer, 2 μL of reverse primer, 2 μL of template and 4 μL of nuclease-free water (Ambion, Austin, TX, USA). For no-template control reactions (NTCs), cDNA was not added and the corresponding 2 μL volumes were allocated to plain nuclease-free water complete with primers and master mix. The loaded well plate was placed onto a StepOnePlus™ Real-Time PCR System (Applied Biosystems) and 40-cycle reactions were performed, complete with melting curves to ensure signals were a consequence of correct primer hybridisation. Results were extrapolated and analysed using the ΔΔC_T_ method. Experiments were performed in triplicates.

## Figures and Tables

**Table 1:** Minimal inhibitory concentrations (MICs) expressed in μg/mL of select MC4 family of nusbiarylins and control drugs used in this work against both methicillin-sensitive and resistant S. aureus strains cultured in TSB.

Compd.	25923	29213	USA300	ST22
**MC4**	1	8	8	4
**MC4-59**	4	4	8	4
**MC4-61**	8	8	8	8
**MC4-72**	8	4	8	8
**Van**	2	2	2	1
**Oxa**	1	1	>64	>64
**Rif**	0.0079	0.0079	0.0079	0.0039

25923: *S. aureus* ATCC^®^ 25923; 29213: *S. aureus* ATCC^®^ 29213; USA300: CA-MRSA strain USA300; ST22: HA-MRSA epidemic clone ST22; Van: vancomycin; Oxa: oxacillin; Rif: rifampicin.

**Table 2:** Primers used for qPCR assays.

Primer	Sequence	Reference
16S_rRNA_F1	GTAGGTGGCAAGCGTTATCC	[57]
16S_rRNA_R1	CGCACATCAGCGTCAG	[57]
PAN23S-F	TCGCTCAACGGATAAAAG	[58]
PAN23S-R	GATGAACCGACATCGAGGTGC	[58]
gyrA-F	CTGAGCGTAATGGTAATGTTGTATG	[59]
gyrA-R	TGCATCTTCTTTTACTTTAGCAACC	[59]
gyrB.MB-F2	CGCAGGCGATTTTACCATTA	[60]
gyrB.MB-R2	GCTTTCGCTAGATCAAAGTCG	[60]
gmk-1	TCGTTTTATCAGGACCATCTGGAGTAGGTA	[61]
gmk-2	CATCTTTAATTAAAGCTTCAAACGCATCCC	[61]
RNAII-11	TATGAATAAATGCGCTGATGATATACCACG	[61]
RNAII-12	TTTTAAAGTTGATAGACCTAAACCACGACC	[61]
RNA3.MB-F	GCCATCCCAACTTAATAACCA	[60]
RNA3.MB-R	TGTTGTTTACGATAGCTTACATGC	[60]
agrA (F)	TGATAATCCTTATGAGGTGCTT	[59]
agrA (R)	CACTGTGACTCGTAACGAAAA	[59]
hla (F) *	GGGGACCATATGATAGAGATT	[59]
hla (R) *	TGTAGCGAAGTCTGGTGAAA	[59]
hla-3 forward **	TGGCCTTCAGCATTTAAGGT	[48]
hla-3 reverse **	CAATCAAACCGCCAATTTTT	[48]
lukS forward	TGAGGTGGCCTTTCCAATAC	[48]
lukS reverse	CCTCCTGTTGATGGACCACT	[48]
spa-F	CAGATAACAAATTAGCTGATAAAAACAT	[59]
spa-R	CTAAGGCTAATGATAATCCACCAAATAC	[59]
sarA_F4	TCTTGTTAATGCACAACAACGTAA	[62]
sarA_R4	TGTTTGCTTCAGTGATTCGTTT	[62]

* Exclusively used for transcripts derived from MRSA strain USA300; ** Exclusively used for transcripts derived from *S. aureus* ATCC^®^ 25923.

## Abbreviations

MRSA	Methicillin-resistant Staphylococcus aureus
MSSA	Methicillin-susceptible *Staphylococcus aureus*
HA-MRSA	Hospital-acquired methicillin-resistant *Staphylococcus aureus*
CA-MRSA	Community-associated methicillin-resistant *Staphylococcus aureus*
PVL	Panton–Valentine Leukocidin
Nus	*N*-utilisation substances
RNAP	RNA polymerase
rRNA	Ribosomal RNA
PPI	Protein-protein interaction
MIC	Minimum inhibitory concentration
CLSI	Clinical & Laboratory Standards Institute
ATCC^®^	American Type Culture Collection
MHB	Mueller-Hinton broth
CA-MHB	Cation-adjusted Mueller-Hinton broth
TSB	Tryptic soy broth
CFU	Colony-forming unit
SDS-PAGE	Sodium dodecyl sulphate–polyacrylamide gel electrophoresis
DEPC	Diethyl pyrocarbonate
RBC	Red blood cell
OD_XXX_	Optical density at XXX nm wavelength
qPCR	Quantitative polymerase chain reaction
cDNA	Complementary deoxyribonucleic acid
PBS	Phosphate-buffered saline
